# Time to complementary feeding initiation and its predictors among children aged 9–23 months in Meket District, Northeast Ethiopia: a Cox Weibull regression

**DOI:** 10.1017/jns.2023.71

**Published:** 2023-09-12

**Authors:** Sileshi Fekadu Alemu, Aregash Abebayehu Zerga, Reta Dewau Yimer, Sisay Eshete Tadesse

**Affiliations:** 1 Meket District Health Office, Meket, North Wollo, Ethiopia; 2 Department of Nutrition and Dietetics, School of Public Health, College of Medicine and Health Sciences, Wollo University, Dessie, Ethiopia; 3 Department of Epidemiology and Biostatistics, School of Public Health, College of Medicine and Health Sciences, Wollo University, Dessie, Ethiopia

**Keywords:** Children aged 9–23 months, Complementary feeding and time to initiation

## Abstract

.Globally and nationally, only 64⋅5 and 49⋅2 % of infants received solid or semi-solid foods, respectively. The available evidence indicates that the time to initiate complementary feeding practices is still poor and varies by region. The aim of the present study was to assess the time to initiation of complementary feeding and its predictors among children aged 9–23 months in Meket District, Amhara Region, Ethiopia. A community-based retrospective cohort study was conducted from June to July 2022 among 459 systematically selected mothers/caregivers with their children from 9 to 23 months of age. The result of descriptive statistics was reported by table, frequency, Kaplan-Meier curve and percent. The proportional hazard model assumption was checked, and a Weibull regression model was used to see the predictors of timely initiation of complementary feeding. An adjusted hazard ratio with a 95 % confidence interval and a *P*-value of 0⋅05 were used to declare the significant predictors. The median time of complementary feeding initiation was 6 months. Attending primary education (adjusted hazard ration (AHR) 1⋅8; 95 % CI 1⋅16, 2⋅78), occupation of the mother (AHR 1⋅43; 95 % CI 1⋅04, 1⋅95), home delivery (AHR 1⋅61; 95 % CI 1⋅09, 2⋅37) and birth preparedness (AHR 1⋅37; 95 % CI 1⋅03, 1⋅81) were the predictors of time to complementary feeding initiation. The median time to complementary feeding initiation was consistent with the WHO recommendation. Maternal education, maternal employment, place of delivery and birth preparedness were the predictors of time to initiation of complementary feeding. Therefore, working with the education sector, increasing the delivery rate in health facilities, strengthening counselling on birth preparation, increasing maternity leave until 6 months of age and initiating corner feeding should be part of the complementary feeding practices promotion agenda.

## Introduction

The World Health Organization (WHO) recommends the initiation of complementary foods at 6 months of age. From 6 months on, breast milk alone is insufficient to meet the energy and nutrient requirements of infants and young children^(1)^. To meet this increased demand and promote normal growth and development, infants and young children should obtain additional meals as desired. Globally, only 64⋅5 % of infants aged 6–8 months are fed solid, semi-solid or soft foods^(2)^. In South Asia, 57⋅4 % of children aged 6–23 months were introduced to complementary foods on time^(3)^. In Ethiopia, the prevalence of age-appropriate complementary foods is 49⋅2 %^(4)^. This inadequate complementary food in terms of quality, quantity and frequency of meals is responsible for growth faltering^(5,6)^. In children under the age of five, both early and delayed introductions of complementary foods are associated with poor nutritional status and increased morbidity^(7)^.

Child malnutrition remains one of the most serious public health issues of the twenty-first century, particularly in low- and middle-income countries^(8)^. It is responsible for one-third of under-five mortality^(4)^. Over two-thirds of these deaths are associated with inappropriate child feeding practices^(9)^. Providing age-appropriate, optimal complementary feeding will help to prevent 6 % of deaths among children under the age of five^(10)^. Inappropriate child feeding practices are associated with short adult stature, impaired intellectual development, reduced economic productivity and low offspring birth weight^(11)^.

Several studies revealed that delayed initiation of complementary feeding is caused by breast problems such as sore nipples or the mother's perceptions of milk production, parental employment status, length of maternity leave, poor knowledge of breast-feeding, mothers’ educational status, fathers’ occupation, place of delivery, birth preparedness, antenatal care (ANC) and postnatal care (PNC) follow-up, the child's sex, having growth monitored, and husband support.

Despite the implementation of infant and young child feeding practice guidelines, undernutrition is still a public health problem in Ethiopia. The rate of decline in undernutrition is not on track to meet the Sustainable Development Goal^(12)^. In low-income countries, appropriate complementary feeding practices remain a challenge for most households, with slow progress^(8)^. The majority of previous studies were cross-sectional in nature, making it impossible to show the median time or cause-and-effect relationships. There is also inconsistency in the proportion and contributing factors of complementary feeding initiation^(13–15)^. In spite of search efforts, no study was documented in the study area. Therefore, the aim of the present study was to fill the gaps left by previous studies by determining the time to initiation of complementary feeding and its predictors among children aged 9–23 months in Meket district. The findings of the study will assist program implementers and stakeholders in making evidence-based decisions for improving the health of children by promoting age-appropriate child feeding practices.

## Methods and materials

### Study area

This study was conducted in Meket District, North Wollo Zone. The district is found at a distance of 660 km to the northeast of the capital city of Ethiopia, Addis Ababa, and about 220 km east from the capital of the Amhara regional state, Bahir Dar. Geographically, it is found at an altitude of 700–2750 metres above sea level. The district has 36 Kebeles and a total population of 214 093, out of which 50⋅8 % are males and 49⋅2 % are females. Among these 9397 are mothers who have children from 9 to 23 months of age^(16)^.

### Study design and period

A community-based retrospective cohort study was carried out from 20 June to 20 July 2022.

### Source population

All children aged 9–23 months who lived in Meket district with their mothers/caregivers were the source population.

### Study population

The study population was all children aged 9–23 months who lived in the selected Kebeles of Meket district with their mothers/caregivers during the data collection period.

### Inclusion and exclusion criteria

Mothers/caregivers having children between 9 and 23 months of age who lived at least 6 months in the district were included in the study. Mothers who were critically ill during the data collection period and unable to recall the initiation time were excluded.

### Sample size determination

The sample size was determined using Epi Info version 7 by considering the following assumptions: 95 % confidence level, 80 % power, non-exposed to exposed ratio of 1 and outcome variable among non-exposed of 57⋅02^(17)^. As a result, after accounting for a design effect of 1⋅5 and a non-response rate of 10 %, the final sample size was 459.

### Sampling technique

Out of 36 Kebeles in the district, eleven were selected and included in the study by a lottery method. The number of study participants was allocated for each Kebele based on population proportion size (PPS). Before data collection, a sampling frame was developed based on the Health Extension Workers’ Registry Book. Then, children–mother/caregivers pair with their house numbers were selected from the registry book. Then, data collectors selected the child–mother/caregivers pair, along with their house numbers, through house-to-house visits in each Kebele using a simple random sampling technique and included them in the study.

### Operational definition

#### Time to initiate CF

The time at which the mother/caregiver starts providing the child with either solid, semi-solid or liquid foods other than breast milk or formula feeding^(14)^.

#### Right censoring

It occurs when the event in question has not yet occurred at the time of last observation or has been lost to follow-up or death prior to the outcome of interest.

#### Left censoring

The event of interest took place at an unknown time prior to the actual observed time.

#### Good knowledge

Respondents were categorised as having ‘good knowledge’ on initiation of complementary feeding (CF) if they scored above or equal to the mean knowledge score^(17)^.

*Poor knowledge:* Respondents were categorised as having poor knowledge upon initiation of CF if they scored below the mean score on six CF knowledge questions^(17)^.

*Growth monitoring and promotion:* Participation of a child in growth monitoring and promotion (GMP) services at least once for 0 months, at least two times for 1–3 months, at least five times for 4–11 months and at least four times per year for 12–23 months^(18)^.

### Data collection tools and procedures

Data were collected through face-to-face interviews using an interviewer-administered questionnaire adapted from different literatures^(13,14,19)^. Ten diploma nurses were recruited as data collectors, and two BSc holders served as supervisors. The questionnaire consists of socio-demographic characteristics, reproductive health and health service-related factors, a wealth index and knowledge- and practice-related characteristics.

### Control of data quality

The questionnaire was translated to Amharic (the local language), and it was then back translated to the English version by another person to ensure consistency. Two days of training were given for data collectors and supervisors on the overall data collection process. The questionnaire was pretested on 5 % of the sample size in Gashena Kebele. Data collectors were closely supervised by supervisors and investigators during the data collection period. The complementary feeding initiation time was ascertained by relating it to known public events like holy days, occurrences of common childhood developmental milestones and immunisation schedules.

### Data analysis

Data entry was done by Epi Info version 7 and exported to STATA version 14 for analysis. The result of descriptive statistics was reported using a table, graph, frequency and percent. The life table was used to estimate the probability of complementary feeding initiation over time, and the Kaplan-Meier (KM) survival curve together with the log-rank test were used to investigate the experience of CF initiation.

The Cox proportional hazards model assumption was checked by a log–log plot of survival. In each of the following survival curves, the graphs crossed each other. This implies that the Cox proportional hazard assumption was not fulfilled. Since the classical survival model is unable to handle the data, the extension of the classical survival model, i.e. Gompertz baseline hazard, was used to report the findings of the study.

The univariate analysis was fitted for every covariate using different baseline distributions, i.e. Weibull, Gompertz, exponential, log-logistic and log-normal. Under multivariable survival analysis, the analysis was done by considering the baseline hazard function and two frailty distributions (Gamma and Inverse-Gaussian).

Variables having a *P*-value of 0⋅25 in the bivariable Cox regression model were transferred to the multivariable Cox regression model. The adjusted hazard ratio with its 95 % CI and a *P*-value of ≤0⋅05 were considered predictors of time to complementary feeding initiation.

Model fitness was checked by the log-likelihood, Akaike information criteria (AIC) and Bayesian information criteria (BIC) values (1). Lower values indicate the goodness of fit of the model. The smallest values of log-likelihood, AIC and BIC were observed in the univariate frailty model with an inverse-Gaussian distribution (log-likelihood = 373⋅06, AIC = 772⋅1 and BIC = 820⋅9). Based on log-likelihood, AIC and BIC values, Gompertz with an inverse-Gaussian shared frailty model was found to be the best model to describe the time-to-death of under-five children in Ethiopia.

### Ethical considerations

The thesis was reviewed and approved by the Institutional Review Board of College of Medicine and Health Sciences, Wollo University, with reference number RCSPG/187/14. A support letter was obtained from Meket District Health Office. This research was conducted based on the Helsinki Declaration. Written informed consent was obtained from the parent or guardian of each participant under 18 years of age, and confidentiality was maintained throughout the study. The data collectors informed the clients that they have the full right to discontinue or refuse to participate in the study.

## Results

### Socio-demographic characteristic

A total of 459 lactating women with their children were interviewed, making the response rate 100 %. The average age of mothers/caregivers was 31⋅18 ± 7⋅87 (sd) years. As shown in Supplementary Table S1, 388 (84 %) of the respondents were rural residents. A large number, 410 (89⋅32 %) of the study participants were Orthodox Christian followers. More than one-third (39⋅43 %) of the respondents had only a primary education. Nearly one-third (34⋅84 %) of husbands had a primary education. Housewives made up two-thirds (66⋅88 %) of mothers and caregivers. More than half 342 (52⋅72 %) of the children were male. Two hundred nine (45⋅53 %) of the respondents were teenagers (Supplementary Table S1).

### Reproductive health-related factors

The majority of pregnancies (404, or 88⋅02 %) were desired by the mother or caregiver. More than three-quarters (366, (79⋅74 %)) of mothers/caregivers had ANC follow-up. A large number, 398 (86⋅71 %) of them were delivered in health facilities, and 381 (83 %) of their mothers had birth preparedness. Three-fourths (76⋅03 %) of mothers/caregivers had growth monitoring and promotion for their children. Three hundred ninety-five (86⋅1 %) of them got child feeding practice counselling during ANC and PNC (Supplementary Table S2).

### Source of information about complementary feeding

The majority of mothers and caregivers (392, or 85⋅5 %) have heard of complementary feeding. Four hundred and two (87⋅58 %) of mothers/caregivers initiated complementary feeding on time. Approximately 403 (or 87⋅8 %) of those polled had heard of exclusive breastfeeding (EBF). One hundred and seventy-seven (43⋅92 %) of them got information about complementary feeding from health extension workers. One hundred and seventy-two (42⋅68 %) of mothers/caregivers obtained information from health professionals, while others obtained it from radio or television.

*Child feeding practices of mothers/caregivers:* The majority of mothers/caregivers (91⋅5 %) were aware of the onset of complementary feeding, and 242 (52⋅3 %) of them initiated at 6 months. Slightly less than half (48⋅15 %) of the respondents fed solid, semi-solid or liquid foods for their children. More than half (58⋅82 %) of mothers/caregivers fed their children three times per day. More than three-fourths (82⋅35 %) of mothers/caregivers wash their hands before feeding their child (Supplementary Table S3).

### Time to complementary feeding initiation among children aged 9–23 months

**Fig. 1.**
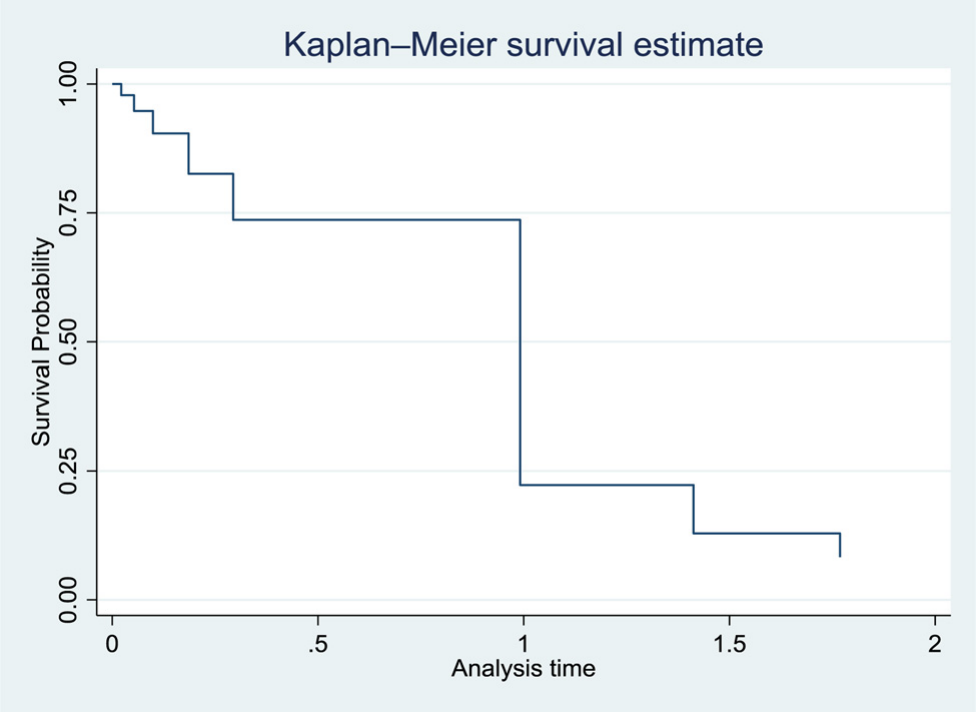
Kaplan-Meier curve showing the survival time to initiation of complementary feeding among children aged 9–23 months old in Meket District, Northeast Ethiopia, July 2022.

The median time for the initiation of complementary feeding was 6⋅00 months (95 % CI 5⋅46, 6⋅60 months). Almost half of (52⋅3 % [95 % CI 52⋅65, 53⋅82]) them initiated complementary feeding appropriately. While 26⋅14 % [95 % CI 22⋅37, 30⋅42] and 21⋅13 % [95 % CI 17⋅53, 24⋅98] began complementary feeding early and late, respectively (Fig. 1).

### Predictors of time to complementary feeding initiation among children aged 9–23 months

**Fig. 2.**
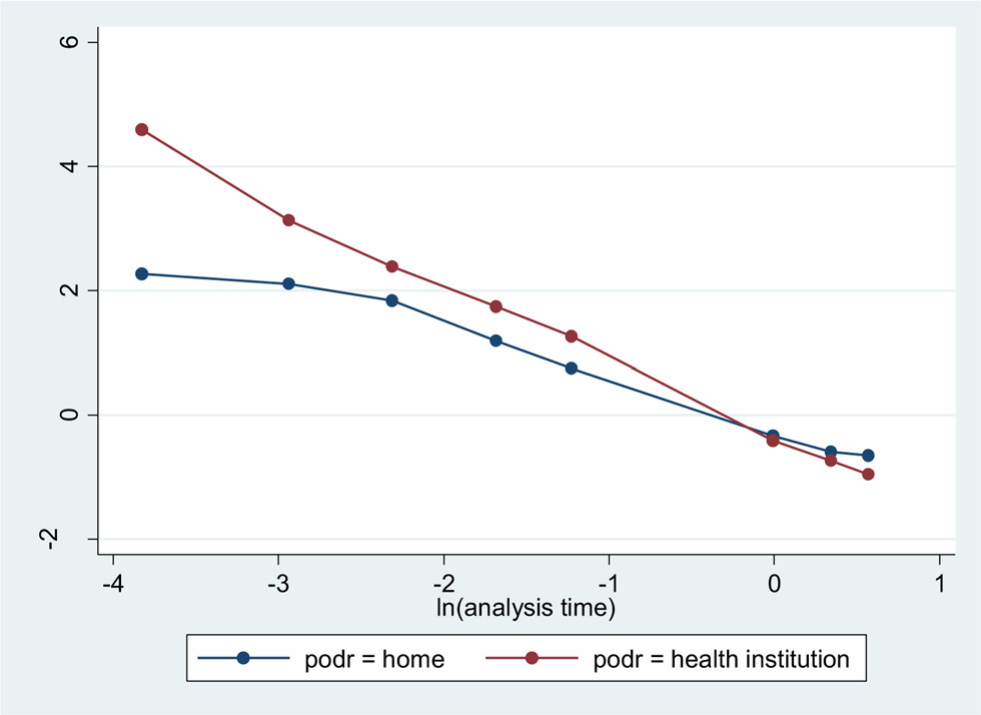
Kaplan-Meier survival curves comparing time to initiation of complementary feeding by place of delivery at Meket District, Northeast Ethiopia, 2022.

The multivariable Weibull regression model revealed that maternal educational status (AHR 1⋅74; 95 % CI 1⋅28, 2⋅38), mother's occupation (AHR  1⋅93; 95 % CI 1⋅27, 2⋅94), birth preparedness (AHR  0⋅72; 95 % CI 0⋅55, 0⋅97) and place of delivery (AHR  0⋅62; 95 % CI 0⋅42, 0⋅91) were found to be predictors of timely initiation of complementary feeding (Supplementary Table S4). The KM survival curve showed that mothers who delivered in a health institution had a better initiation time than mothers who delivered at home (Fig. 2).

**Fig. 3.**
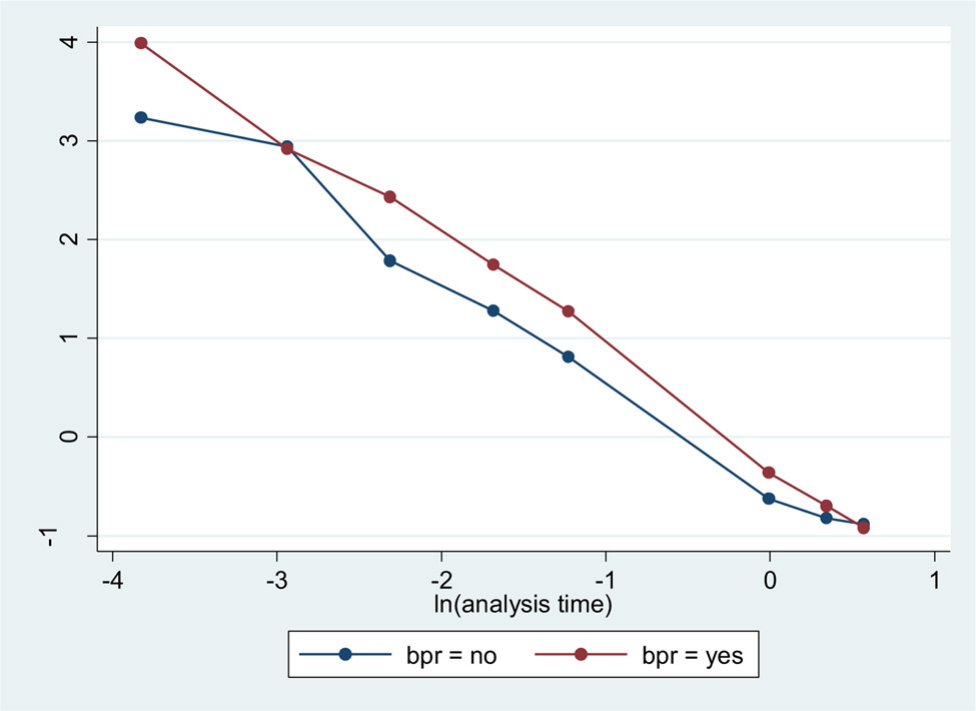
Kaplan-Meier survival curves comparing initiation time between mothers by category of birth preparedness at Meket District, Northeast Ethiopia, 2022.

The KM survival curve showed that mothers with birth preparedness had a better initiation time than mothers with no birth preparedness (Fig. 3).

## Discussion

The aim of the present study was to assess the time to complementary feeding initiation and its predictors among children aged 9–23 months. In the present study, the median age of complementary feeding initiation was 6 months. This result is consistent with studies conducted in Mekelle^(18)^ and Maichew^(14)^. However, this finding was higher than studies conducted in five European countries, Australia and China^(18–20)^. This might be because in developed nations, most mothers do not have enough time to adhere to the WHO recommendation of timely complementary feeding initiation. While women in the study area have enough time to comply with the timely initiation of complementary feeding and get plenty of information on child feeding practices.

This study showed that occupation was found to be a predictor of timely initiation of complementary feeding. The hazard of complementary feeding initiation among government employed mothers was 57 % higher than among housewives. This result was similar to previous studies reported from Maichew, Lasta, Addis Ababa and Kamba district^(14,21–23)^. This could be because the low maternity leave given to government employed mothers and their perception of insufficient milk production did not allow them practice the age-appropriate optimal child feeding practices. On the other hand, studies done in Halaba Kulito town and Nepal contradict this finding^(24,25)^. This inconsistency could be due to the socio-demographic characteristics, sample size and differences in the study setting.

Maternal educational status was found to be a predictor of time to initiation of complementary feeding. The hazard of complementary feeding initiation among mothers who attend primary education was 26 % higher as compared to mothers who attend college and above. This finding is in line with studies conducted in Saudi Arabia^(26)^, Poland^(27)^ and Kamba district^(23)^. This may be due to the fact that maternal education will increase their understanding of the benefits of optimal child feeding practices. The better they are educated, the better they will know about the importance of complementary feeding practices. But a study conducted in Nigeria was inconsistent with this study, where education has no relationship with timely initiation of complementary feeding^(28)^. This is likely to be related to the need to reduce the frequency of breast-feeding and wean early.

Mothers who gave birth in a health facility were more likely to initiate timely complementary feeding as compared to mothers who delivered at home. The risk of timely complementary feeding initiation was 38 % lower in mothers who gave birth in a health facility than in mothers who gave birth at home. This finding is in line with studies conducted in Kenya^(29)^, Axum^(15)^, Addis Ababa^(22)^ and Bishoftu^(30)^. This could be due to the support, advice and nutrition education given by healthcare providers, which will increase their knowledge of age-appropriate complementary feeding.

The risk of complementary feeding initiation was lower among mothers who had birth preparation compared to their counterparts. The hazard of timely complementary feeding initiation among mothers with birth preparedness was 28 % lower than that of mothers who did not have birth preparedness. This finding is supported by studies from Maichew^(14)^ and Mekelle^(17)^. This may be due to the planning they did, which allowed them to put together the circumstances that would allow them to begin complementary feeding on time. Achieving age-appropriate complementary feeding practices has been linked to decreased undernutrition, increased cognitive development, school performance and productivity, and is the foundation for personal development. Recall bias may affect the result of this study.

## Conclusion

In the present study, the median time to complementary feeding initiation was consistent with the national and international recommendations. Maternal educational status, maternal occupation, place of delivery and birth preparedness were the predictors of time to complementary feeding initiation. Therefore, promotion of complementary feeding initiation should include addressing illiteracy through youth education, extending maternity leave to 6 months of age and starting corner feeding and strengthening counselling on skilled delivery and birth preparedness.

## Supporting information

Alemu et al. supplementary material Alemu et al. supplementary material

## Supplementary material

The supplementary material for this article can be found at https://doi.org/10.1017/jns.2023.71.
