# Supplementary material for: Time to complementary feeding initiation and its predictors among children aged 9–23 months in Meket District, Northeast Ethiopia: a Cox Weibull regression
Source: J Nutr Sci. 2023 Sep 12;12:e98. doi: 10.1017/jns.2023.71 (PMC10511819; doi:10.1017/jns.2023.71)
Supplement: Alemu et al. supplementary material [file S204867902300071Xsup001.docx]

Table 1:-Socio demographic characteristics of respondents in Meket district, Northeast Ethiopia, July 20, 2022

| Variable | Category | Frequency | % |
| --- | --- | --- | --- |
| Age of the mother | 14-24 years | 96 | 20.92 |
|  | 25-34 years | 187 | 40.74 |
|  | 35 and above | 176 | 38.34 |
| Residence of mother | Rural | 388 | 84.53 |
|  | Urban | 71 | 15.47 |
| Marital status | Married | 396 | 86.27 |
|  | Divorced | 11 | 2.39 |
|  | Single | 17 | 3.70 |
|  | Widowed | 35 | 7.63 |
| Educational status of Husbands | Illiterate | 132 | 32.84 |
|  | Primary school | 138 | 34.33 |
|  | Secondary school | 81 | 20.15 |
|  | College and above | 51 | 12.69 |
| Occupation of Husbands | Farmer | 221 | 55.53 |
|  | Merchant | 87 | 21.86 |
|  | Private employee | 41 | 10.30 |
|  | Government employee | 49 | 12.31 |
| Maternal educational status | Illiterate | 98 | 21.35 |
|  | Primary school | 182 | 39.43 |
|  | Secondary school | 138 | 30.07 |
|  | College and above | 42 | 9.15 |
| Maternal occupation | House wife | 307 | 66.88 |
|  | Merchant | 72 | 15.69 |
|  | Private employee | 40 | 8.71 |
|  | Government employee | 40 | 8.71 |
| Religion | Orthodox | 410 | 89.32 |
|  | Protestant | 11 | 2.20 |
|  | Muslim | 38 | 8.28 |
| Sex of the child | Male | 242 | 52.72 |
|  | Female | 217 | 47.28 |
| Age of the child | 9-13 months | 142 | 30.94 |
|  | 14-18 months | 209 | 45.53 |
|  | 19-23 months | 108 | 23.53 |
| Wealth index | Poor | 170 | 43.9 |
|  | Middle | 113 | 29.2 |
|  | Rich | 104 | 26.9 |

Table 2: Reproductive health and health service utilization related characteristics of respondents in Meket district, Northeast Ethiopia, July 20, 2022

| **Variables** | **Category** | **Frequency** | **%** |
| --- | --- | --- | --- |
| Types of pregnancy | Wanted | 404 | 88.02 |
|  | Unwanted | 55 | 11.98 |
| ANC follow up | Yes | 366 | 79.74 |
|  | No | 93 | 20.26 |
| Place of delivery | Health institution | 398 | 86.71 |
|  | Home | 61 | 13.29 |
| Mode of delivery | Vaginal delivery | 269 | 72.14 |
|  | Cesarean section | 100 | 27.86 |
| Birth preparedness | Yes | 381 | 83.01 |
|  | No | 78 | 16.99 |
| Growth monitoring | Yes | 349 | 76.03 |
|  | No | 110 | 23.97 |
| Number of children | <3 children | 304 | 66.23 |
|  | 4-6 children | 145 | 31.59 |
|  | > 6 children | 10 | 2.18 |
| Family size | <3 | 74 | 16.12 |
|  | 4-6 | 284 | 61.87 |
|  | ≥7 | 101 | 22.00 |
| Post-natal care | Yes | 368 | 80.17 |
|  | No | 91 | 19.83 |
| Getting counseling during ANC/PNC follow up | Yes | 395 | 86.1 |
|  | No | 64 | 13.9 |

Table 3: Child feeding practices in Meket district, Northeast Ethiopia, July 20, 2022.

| **Variable** | **Category** | **Frequency** | **%** |
| --- | --- | --- | --- |
| Initiated complementary feeding | Yes | 420 | 91.5 |
|  | No | 39 | 8.5 |
| Time of initiation | Before 6 months | 120 | 26.14 |
|  | At 6 months | 242 | 52.72 |
|  | After 6 months | 97 | 21.13 |
| Frequency of feeding | 1-2 times/day | 107 | 25.48 |
|  | 3 times/day | 185 | 44.05 |
|  | 4 times/day | 128 | 30.48 |
| Ways of feeding the children | Spoon | 158 | 37.62 |
|  | Hand | 163 | 38.81 |
|  | Bottle | 53 | 12.62 |
|  | Cup | 46 | 10.95 |
| Cause of early initiation of CF | Maternal medical illness | 26 | 32.5 |
|  | Lack of information | 21 | 26.25 |
|  | Distance of working place to home | 23 | 28.75 |
|  | Elderly or family advice | 10 | 12.50 |
| Hand washing practice | Yes | 378 | 82.35 |
|  | No | 81 | 17.65 |
| Maternal medical illness during PNC | Yes | 107 | 23.31 |
|  | No | 352 | 76.69 |

Table 4: Determinants of complementary feeding initiation among mothers with children age 9 to 23 months in Meket District North Wollo Zone, July 20, 2022

| **Variables** | **Category** | **Timely initiation of CF** | | **CHR(95%CI)** | **AHR(95%CI)** |
| --- | --- | --- | --- | --- | --- |
|  |  | **Yes (%)** | **No (%)** |  |  |
| Age of the mother | 14-24 years  25-34 years  35 and above | 80(83.33)  173(92.51)  167(94.89) | 16(16.67)  14(7.49)  9(5.11) | 1  1.36(1.04-1.77)  1.53(1.17-2.01) | 1  1.26(0.92-1.7)  1.34(0.98-1.82) |
| Maternal educational status | Illiterate  Primary school  Secondary school  College and above | 88(89.80)  172(95.03)  123(89.13)  37(88.1) | 10(10.20)  9(4.97)  15(10.87)  5(11.9) | 1.00(0.68-1.47)  1.66(1.16- 2.37)  1.10(0.76- 1.58)  1 | 1  **1.74 (1.28, 2.38) ***  1.33 (0.96, 1.86)  0 .83 (0 .49, 1.39) |
| Maternal occupation | House wife  Merchant  Government employed | 285(92.83)  63(87.5)  72(90.0) | 22(7.17)  9(12.5)  8 (10.0) | 1  0.71(0.54-0.94)  0.95(0.73-1.23) | 1  0.79 (0.57-1.11)  **1.43 (1.04-1.95)**** |
| ANC follow up | Yes  No | 337(92.08)  83(89.25) | 29(7.92)  10(10.75) | 1  0.76(0.59-0.96) | 1  0.80(0.6-1.08) |
| Place of delivery | Health institution  Home | 368(92.69)  52(83.87) | 29(7.31)  10(16.13) | 0**.81(0.6-1.08**)  1 | **0.62 (0.42, 0.91) ****  **1** |
| Birth preparedness | Yes  No | 349(91.56)  71(91.03) | 32(8.44)  7(8.97) | 1.18(0.91, 1.52)  1 | **0.72 (0.55, 0 .97)****  **1** |
| Counselling during PNC follow up | Yes  No | 365(92.41)  55(85.94) | 30(7.59)  9(14.06) | 1  0.84(0.63-1.12) | 1  0.68(0.29-1.57) |
| Information of CF | Yes  No | 364(82.86)  56(83.52) | 28(7.14)  11(16.42) | 1  0.75(0.57- 1.00) | 1  1.36(0.48-3.88) |
| Information of right time of initiate CF | Yes  No | 373(92.8)  47(82.46) | 29(7.2)  10(17.54) | 1  0.77(0.57-1.04) | 1  1.05(0.25-4.46) |

* Indicate Significant a p value< 0.001, ** Significant a p value< 0.05
